# Clinical Characteristics and Gene Mutation Analysis of the Chinese Han Population with Gitelman Syndrome: 3 Case Reports and a Literature Review

**DOI:** 10.1155/2020/6263721

**Published:** 2020-10-24

**Authors:** Xueting Li, Ruofei Chen, Mingwei Chen

**Affiliations:** ^**1**^ Department of Endocrinology, The First Affiliated Hospital of Anhui Medical University, Hefei, Anhui 230032, China; ^**2**^ Department of Rheumatology and Immunology, The First Affiliated Hospital of Anhui Medical University, Hefei, Anhui 230032, China

## Abstract

The present study reported clinical characteristics and the results of gene mutation analysis of 3 Chinese patients with Gitelman syndrome (GS). Three patients manifested with normal blood pressure, recurrent hypokalemia, and metabolic alkalosis. Only case 2 had obvious hypomagnesemia. Gene sequencing showed a compound heterozygous mutation in SCL12A3 in case 1 and a homozygous mutation in SCL12A3 in case 2. Heterozygous mutations in SCL12A3 and CLCNKB were found in case 3. Then, the literature was reviewed. The keyword “Gitelman syndrome” was inputted into the PubMed, Wanfang Database, and CNK to search all Chinese patients with GS diagnosed by gene mutations and to extract complete clinical data from December 1998 to 2018. Finally, a total of 124 cases of GS were included. No significant differences in the levels of serum potassium and magnesium were observed among the different gene mutations, and the serum magnesium levels in adults were lower than those of the juvenile. GS with reduced blood magnesium had a serious clinical phenotype. Therefore, GS had a diverse phenotype, and its final diagnosis required genetic profiling. The relationship of gene mutation and clinical phenotype needed further study.

## 1. Introduction

Gitelman syndrome (GS) is an autosomal recessive renal tubular disease, first reported by Gitelman et al. in 1966 [1]. GS is caused by a mutation in the SLC12A3 gene which encodes a thiazide-sensitive sodium chloride cotransporter (NCCT) [2, 3]. CLCNKB mutations form the molecular basis of classical Bartter syndrome (cBS) as it encodes chloride channels in the renal tubular basement membrane [4]. It was recently reported that CLCNKB mutations also cause GS [2, 5]. In this study, 36 hypokalemia-related genes were sequenced and analyzed by high-throughput second-generation sequencing in 3 patients with hypokalemia. We aimed to identify gene mutation sites that could improve disease diagnosis and treatment. We also searched full-text journals published in the PubMed, WanFang Database, and China Knowledge Network (CNK) to summarize the relationship between clinical data and mutation types.

## 2. Case Presentation

All 3 patients showed renal potassium loss, metabolic alkalosis, high renin activity, but normal blood pressure. The patients did not take long-term laxatives, diuretics, and other drugs, excluding secondary factors such as hyperthyroidism, primary aldosteronism, renal artery stenosis, cortisol hypertrophy, renin tumors, or tubular acidosis.

### 2.1. Case 1

A 23-year-old male patient was hospitalized for hypokalemia since 2 years. Two years earlier, the patient had hypokalemia due to severe diarrhea after eating unclean food. No fatigue, numbness of limbs, palpitation, or chest tightness was noted. Serum potassium was 2.77 mmol/L during a routine physical examination. The patient developed normally and is unmarried. There was no history of similar disease in the family. Consanguineous marriage was denied. After admission, a physical examination was performed: blood pressure, 110/70 mmHg. The results of electrolytes at admission showed that the levels of serum potassium and serum magnesium were 2.84 mmol/L and 0.77 mmol/L, respectively. Blood gas analysis showed mild metabolic alkalosis.

### 2.2. Case 2

A 41-year-old male patient was hospitalized due to elevated blood sugar levels that persisted for half a year. The patient suffered from hypokalemia for 8 years (blood potassium 2.5–2.9 mmol/L). The patient suffered from occasional feebleness and aching in both the lower limbs, which improved after potassium supplementation. There was no history of similar disease in the family. Consanguineous marriage was seen in his parents. After admission, a physical examination was conducted: blood pressure, 108/68 mmHg. The results of electrolytes at admission showed that the levels of serum potassium and serum magnesium were 2.39 mmol/L and 0.56 mmol/L, respectively. Blood gas analysis showed metabolic alkalosis.

### 2.3. Case 3

**Figure 1:**
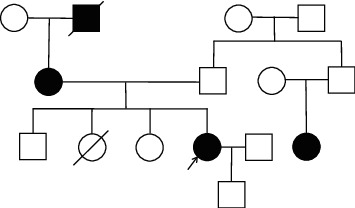
Family tree of the case 3 families who suffer from hypokalemia. Square = male, circle = female.

A 37-year-old female patient was hospitalized for reduplicated twitching limbs for 10 years and syncope on 3 occasions. Ten years earlier, the patient suffered from limb twitching without obvious induction, which was accompanied by numbness and the twitching of both hands. Over the last 10 years, recurrent syncope occurred on three occasions. This was accompanied by sweating, a loss of consciousness, and urine incontinence. The symptoms lasted for several minutes and improved spontaneously, mostly after fatigue. In many emergency cases, the patient's serum potassium level was 2.5–3.0 mmol/L. The 24-hour urinary calcium excretion quantitative value was 2.57 mmol, which was lower than the normal reference value. In addition, the oral hydrochlorothiazide diuretic test (HCT test) was performed in this patient as described by Colussi et al. [6]. The results showed that the maximum difference of chlorine excretion rate before and after HCT test was 2.1%, indicating that there was dysfunction of distal renal tubules. Moreover, the patient was diagnosed with hyperthyroidism and failed to adhere to medication. Her deceased grandfather suffered from limb numbness and was not examined for blood potassium. Her mother suffered from limb numbness and low blood potassium levels (the specific situation is unknown), but her symptoms improved after oral potassium chloride. The elderly brothers and sisters were assessed for blood potassium which was normal on 3 occasions. One elderly sister died, the cause of death being unclear. A cousin displayed numbness of the limbs with blood potassium levels of 2.81 mmol/L. One son had no obvious numbness in his limbs, and his blood potassium was normal. A family tree is shown in Figure 1. Following admission, a physical examination was performed: blood pressure, 126/80 mmHg. The results of electrolytes at admission showed that the levels of serum potassium and serum magnesium were 3.29 mmol/L and 0.79 mmol/L, respectively. Blood gas analysis showed metabolic alkalosis.

There were no abnormal blood lipids in the 3 patients including blood coagulation factors. Liver and kidney function were normal. Amongst the patients, case 2 had diabetes mellitus. The results of blood potassium, magnesium, calcium, 24-hour urine potassium, and blood gas analysis are detailed in Table 1. All 3 patients had hypokalemia, renal potassium loss, and metabolic alkalosis. Thyroid function, adrenal cortex hormones, and aldosterone were normal. Renin levels increased in all 3 cases. In addition, no obvious abnormalities in adrenal CT and pituitary MRI were observed in the 3 patients.

High-throughput second-generation sequencing and bioinformatics analysis of the 36 hypokalemia-related genes were performed to identify possible mutation sites. Direct sequencing was used to verify the patients and their parents (Table 2). In case 1, two compound heterozygous mutations in the SCL12A3 gene were identified. One originated from the father (p. T60M), and the other originated from the mother (p. L858H). Case 2 had a homozygous mutation (p.N359K) from the parents. Case 3 had two genetic mutations from the mother, including the P.T60M mutation in the SCL12A3 and P.W391R mutation in the CLCNKB gene. These are known as Bartter syndrome type III and classic Bartter syndrome mutations.

## 3. Literature Review

We inputted the keyword “Gitelman syndrome” into the PubMed, Wanfang Database, and CNK to search all Chinese articles from December 1998 to 2018. Following screening, 48 papers with detailed and complete patient data were selected. A total of 124 cases were diagnosed as GS by gene mutation analysis. The age of onset ranged from 1 year to 64 years. Amongst the patients, 25 were ≤18 years old. A total of 17 patients had normal serum magnesium.

Amongst the 124 cases, there were 56 compound heterozygous mutations, 28 homozygous mutations, 26 multiple mutations, and 14 single heterozygous mutations. The 124 cases included 17 frameshift mutations and 6 nonsense mutations (Table 3). The detection of urinary potassium was influenced by the levels of blood potassium and whether the patients received supplementary potassium. The reference values of urinary magnesium differed between hospitals. And the difference of aldosterone renin levels was greater. We therefore compared only serum potassium and magnesium levels amongst the mutants.

No significant differences between the 23 cases of frameshift and nonsense mutations and the 101 cases of other mutations in terms of blood potassium and magnesium levels were observed. No significant differences in serum potassium and magnesium levels were observed amongst the groups with compound heterozygous mutations, homozygous mutations, multiple mutations, and single heterozygous mutations. There were no obvious abnormalities in serum potassium levels in the adult or juvenile groups, but the serum magnesium levels in the adult group were lower than those of the juvenile group (Table 4). In addition, the average serum potassium level in the low blood magnesium group was significantly decreased compared with the normal blood magnesium group (*P*=0.003) (Table 5).

## 4. Discussion

GS is characterized by hypokalemia, hypomagnesia, hypocalcemia, metabolic alkalosis, hyperrenin-angiotensin aldosterone, and normal or low blood pressure. It is a common tubular genetic disease caused by mutations in the SLC12A3 gene [1]. The disease is autosomal recessive inheritance, and the symptom rarely occurs before the age of six. GS is mostly diagnosed in adolescence or adulthood, and its general clinical features include muscle weakness and tetany. Mutations in the CLCNKB gene that encode the renal basement chloride channel form the molecular basis of the classical Bartter's syndrome [4]. GS and Bartter's syndrome overlap according to the phenotype. Hypomagnesemia and hypocalciuria are the major features of GS [7], which differs from the classical Bartter's syndrome. However, hypomagnesemia and hypocalciuria are not always present in patients with GS [8]. It has been reported that the CLCNKB mutations in the classical Bartter's syndrome cause cases similar to GS [2, 5].

All 3 cases of hypokalemia were diagnosed in adulthood. Case 1 had no obvious myasthenia, convulsions of the hands and feet, and hypomagnesemia, but only mild metabolic alkalosis. The clinical phenotype was similar to Bartter's syndrome. Case 2 showed occasional weakness and soreness of both lower limbs, mild hypomagnesemia, and hypochloride metabolic alkalosis. The clinical manifestations of case 3 were severe, with obvious weakness of both lower limbs, convulsion and asthenia, and syncope. Following potassium supplementation, the symptoms improved. There was no hypomagnesemia, and the 24-hour urinary calcium excretion quantitative value was significantly decreased. The clinical phenotype was similar to that of Bartter's syndrome. The genetic analysis of cases 1 and 2 were SCL21A3 complex heterozygous mutations and homozygous mutations, respectively. All three mutation sites were previously reported in the literature [9–11]. In case 3, there were two gene mutations, both from the mother. One was the p.t60m mutation in the SCL12A3 gene, and the other was the p.w391r mutation of the CLCNKB gene, which was type of gene mutation of III Barter's syndrome. To date, the p.w391r mutation of the CLCNKB gene represents a newly discovered mutation site and a nonpolymorphic site, the incidence rates of which are low. Moreover, the mutations were not reported in the professional edition of the Human Gene Mutation Database (HGMD). GS and Bartter's syndrome are autosomal recessive inheritance. It was reported that chloride clearance test can effectively differentiate GS and BS [12]. In order to further confirm whether case 3 is GS, we carried out hydrochlorothiazide load test on the patient. The results showed that the chlorine excretion rate did not increase significantly after the administration of hydrochlorothiazide. Therefore, it was preliminarily determined that the patient was GS. But further genome-wide sequencing and multiplexed probe amplification (MLPA) is required to detect large fragment deletions or duplications.

It has been reported that phenotypic differences exist not only in family members but also in patients with the same genetic mutations [13]. Case 3 also had Graves' disease. The thyroid function was normal during hospitalization, and no previous medication was administered. Zhou et al. [14] summarized 17 cases of GS with autoimmune thyroid disease, including 13 cases with Graves's disease. All 13 patients presented with hypokalemic periodic paralysis, the mechanism(s) of the occurrence of which remain unclear.

NCCT is expressed in the distal convoluted tubule of the nephrons and represents the major ion transport system in this region. The SLC12A3 gene is located on chromosome 16q13 and is approximately 55 kb in length with a total of 26 independent exons [2, 3]. The human SLC12A3 gene has been cloned and is predicted to encode a 1021 amino acid protein, that includes 12 transmembrane domains and long amino and carboxy ends. Over 100 mutations are associated with GS [15, 16], including missense mutations, nonsense mutations, coding frameshift mutations, and shear mutations. Meanwhile, homozygous mutations are rare. A large number of heterozygous mutations have been identified. In this study, 36 genes associated with hypokalemia were analyzed by high-throughput sequencing. For cases 1 and 3, complex heterozygous mutations in SCL21A3 were identified. Case 2 was a homozygous mutation. The P.T60M mutation in case 1 is the most common mutation in Asians [13, 17]. The other mutations, p.l858h, were discovered by Japanese researchers in 2012 [10]. Case 2 had a homozygous mutation, and the patient's parents were close relatives. The mutation type was P.N359K which was reported in Chinese studies in 2009 [11]. Case 3 had a heterozygous mutation which was more complex. Mutations of two genes were found, both from the mother, namely, p.t60m of SCL12A3 and p.w391r of CLCNKB. Interestingly, the patient's mother also had hypokalemia. There are currently no reports of simultaneous SCL12A3 and CLCNKB mutations in the literature. Both mutations were heterozygous mutations. In Asia, the SCL12A3 P.T60M and CLCNKB P.W391R mutations are common. The CLCNKB mutation leads to the development of the classical Bartter's syndrome, which has autosomal recessive inheritance. Although the frequency of heterozygous mutations increases with GS, other mutations may be present. The reasons are as follows: (1) the mutation sites may be located in a regulatory sequence of SLC12A3 that we did not sequence, such as 5′ or 3′ untranslated regions or deep introns; (2) single nucleotide polymorphisms (SNPs) may interfere with the expression and function of NCCT; (3) mutations may be located in other genes that regulate NCCT function; and (4) gene expression and function may be acquired [17].

In this study, 124 cases of GS were diagnosed by gene mutational analysis with complete data and are summarized. These include 56 compound heterozygous mutations, 28 homozygous mutations, 26 multiple mutations, 14 single heterozygous mutations, 17 frameshift mutations, and 6 nonsense mutations. No significant differences in the levels of potassium or magnesium between the frameshift and nonsense mutations were observed, similar to previous studies [18]. Data from the Zhongshan Hospital of China showed that complex heterozygotes/homozygotes of the SLC12A3 gene have significantly lower serum potassium levels than the single heterozygotes [19]. In this study, no significant differences in serum potassium and magnesium levels were observed in either group. This may be related to the inconsistency of the detection methods. This article is a summary of an array of cases that used different detection techniques. Some employed multiplexed probe amplification technology (MLPA) detection, resulting in a higher frequency of mutation sites in which the number of compound heterozygous mutations increased. Some of the hospitals used MLPA to detect simple heterozygous mutations with different statistical parameters. This small number of cases now requires expansion to obtain statistically accurate data.

Electrolyte imbalances, acid-base imbalances, and clinical manifestations occur more frequently in patients with low serum magnesium [20, 21]. Amongst the 124 patients assessed in this study, 17 had normal serum magnesium, and the average serum potassium level in the low blood magnesium group was significantly decreased compared with the normal blood magnesium group. This was consistent with the more serious clinical manifestations in patients with low serum magnesium.

GS and the classical Bartter's syndrome are incurable, but the prognosis is promising. However, the ion disorder in GS patients is difficult to treat and requires long-term use of a combination of medications [22]. GS-mediated hypokalemia and metabolic disorders can be corrected with potassium supplements or potassium-sparing diuretics. For severe cases, simultaneous magnesium supplementation is required [21]. The treatment of Bartter's syndrome is to correct hypokalemia and alkalosis. Potassium-preserving diuretics such as spironolactone combined with potassium chloride help to control hypokalemia over a short time period. The most effective treatment is prostaglandin synthase inhibitors such as indomethacin, aspirin, and ibuprofen. In addition, angiotensin-converting enzyme inhibitors can reduce potassium excretion by inhibiting the renin-angiotensin-aldosterone system, which may be superior to spironolactone.

In brief, GS had a diverse phenotype, and its final diagnosis required further genetic mutation analysis. Hypokalemia caused by hereditary diseases should be considered as the possibility of GS. The relationship of gene mutation and clinical phenotype needs further study.

## Figures and Tables

**Table 1:** Serum electrolytes, 24-hour urine potassium, blood gas analysis, and renin aldosterone levels of the 3 cases.

Case	Case 1	Case 2	Case 3	Reference range
Potassium (mmol/L)	2.84	2.39	3.29	3.5∼5.3
Sodium (mmol/L)	140	138	145	137∼147
Chloride (mmol/L)	94	98	101	99∼110
Calcium (mmol/L)	2.47	2.46	2.45	2.00∼2.60
Magnesium (mmol/L)	0.77	0.56	0.79	0.66∼1.07
Potassium of 24-h urine (mmol/24 h)	56	66	34	—
Renin (*μ*g/L^*∗*^H)	7.26	9.5	8.88	0.1∼6.56
Aldosterone (ng/L)	120	156	209	70∼300
Arterial blood gas analyses				
pH	7.43	7.51	7.48	7.35∼7.45
Base excess (mmol/L)	5.2	5.6	7.8	−3∼3
Bicarbonate (mmol/L)	29.1	29.4	31.6	21.3∼24.8

**Table 2:** Gene mutations in the 3 patients.

Case	Gene	Exon	Mutation	Zygote	Effects
Case 1	SCL21A3	1	c.179C > *T*	Complex heterozygous	p.T60M
		22	c.2573T > *A*		p.L858H
Case 2	SCL21A3	8	c.1077C > *G*	Homozygous	p.N359K
Case 3	CLCNKB	12	c.1171T > *C*	Heterozygous	p.W391R
	SCL21A3	1	c.179C > *T*	Heterozygous	p.T60M

**Table 3:** Comparison of blood potassium and blood magnesium levels across the different mutation types ($\bar{x}$ ± *s*).

Group	Number	Potassium (mmol/L)	Magnesium (mmol/L)
Compound heterozygous mutations	56	2.49 ± 0.48	0.55 ± 0.12
Homozygous	28	2.45 ± 0.41	0.59 ± 0.15
Multiple mutations	26	2.51 ± 0.47	0.61 ± 0.18
Single heterozygous mutation	14	2.51 ± 0.51	0.52 ± 0.17
Frameshift and nonsense mutation	23	2.47 ± 0.46	0.55 ± 0.15

**Table 4:** Comparison of serum potassium and magnesium levels in the adult and juvenile groups ($\bar{x}$ ± *s*).

	Number	Potassium (mmol/L)	Magnesium (mmol/L)
Adult	99	2.96 ± 0.35	0.57 ± 0.31
Juveniles	25	2.93 ± 0.42	0.68 ± 0.24
*P* value		0.602	0.034

**Table 5:** Comparison of serum potassium levels between the low blood magnesium and normal blood magnesium groups ($\bar{x}$ ± *s*).

	Number	Potassium (mmol/L)
Low blood magnesium	107	2.42 ± 0.45
Normal blood magnesium	17	2.84 ± 0.44
*P* value		0.003
